# Metacognitive therapy and work-focused interventions for patients on sick leave due to anxiety and depression: study protocol for a randomised controlled wait-list trial

**DOI:** 10.1186/s13063-021-05822-4

**Published:** 2021-11-27

**Authors:** Kenneth Sandin, Ragne G. H. Gjengedal, Kåre Osnes, Marit Hannisdal, Torkil Berge, Jonas S. R. Leversen, Lars G. Røv, Silje Endresen Reme, Suzanne Lagerveld, Roland Blonk, Hans M. Nordahl, Gemma Shields, Adrian Wells, Odin Hjemdal

**Affiliations:** 1 Division of Mental Health and Substance Abuse, Diakonhjemmet Hospital, Postboks 23 Vinderen, 0319 Oslo, Norway; 2 Department of Psychology, Norwegian University of Science and Technology, NO-7491 Trondheim, Norway; 3 Department of Psychology, Faculty of Social Sciences, University of Oslo, Oslo, Norway; 4 Dutch Institute for Employee Benefit Schemes (UWV), Amsterdam, The Netherlands; 5 TNO Leiden, Leiden, The Netherlands; 6 Faculty of Medicine and Health Sciences, Norwegian University of Science and Technology, Trondheim, Norway; 7 Division of Population Health, Health Services Research, and Primary Care, University of Manchester, Manchester, UK; 8 Faculty of Biology Medicine and Health, The University of Manchester, Manchester, UK

**Keywords:** Sick leave, Anxiety, Depression, Work-focused interventions, Metacognitive therapy, Cost-effectiveness, Randomised controlled trial

## Abstract

**Background:**

Common mental disorders such as depression and anxiety are major contributors to the global burden of disease. Affected individuals suffer reduced quality of life, impaired functioning and reduced capacity to work. Maintaining employment is an important determinant for health and wellbeing, and the economic impact of depression and anxiety is a significant societal expense. Treatments providing effective symptom reduction and helping patients return to work (RTW) would thus have substantial public health benefits. The present study will explore the effectiveness of metacognitive therapy (MCT) and work-focused interventions on reducing symptoms and increasing RTW rates for patients on sick leave due to depression and anxiety.

**Methods:**

The study is a randomised controlled wait-list trial (RCT; *N* = 240). The intervention group will receive protocol-based MCT and work-focused interventions immediately after inclusion. The control condition is a wait-list control group. All patients will receive up to 12 weekly sessions. The study context is a Norwegian outpatient clinic part of a national programme aimed at reducing sick leave. The co-primary outcomes are change in RTW and symptoms of depression and anxiety at the end of treatment. In addition to self-report, sick leave will also be collected from national registries from 2 years prior to intervention to 4 years after intervention. Symptoms of depression and anxiety will be collected by self-report at pre- and post-treatment and at 6 and 12 months follow-up after treatment. A cost-effectiveness analysis will use total cost and quality-adjusted life-years as the secondary outcomes.

**Discussion:**

There is broad consensus on the importance of identifying treatment that effectively reduces depression and anxiety symptoms and aids RTW. This study is an important contribution to the field as it is the first RCT on MCT and work-focused interventions for patients on sick leave due to anxiety and depression.

**Trial registration:**

ClinicalTrials.gov NCT03301922. Registered on October 4, 2017.

**Supplementary Information:**

The online version contains supplementary material available at 10.1186/s13063-021-05822-4.

## Background

Recent estimates indicate that mental illness may account for nearly a third (32.4%) of total years lived with disability, making it the main contributor to the global burden of disease [1]. Common mental disorders such as depression and anxiety are the most prevalent mental illnesses and thus account for the largest share of the disease burden [2, 3]. Affected individuals suffer reduced quality of life, including functional impairment [4]. In Europe, depression is responsible for 13.7% of all years lived with disability, and anxiety disorders likely have a similar impact [5, 6]. In 2018, the economic cost of mental disorders in Europe was estimated to be €600 billion a year, 40% of which was due to reduced employment and lost productivity [7].

In addition to the economic cost, loss of employment is detrimental to health and wellbeing, and work impairment is associated with negative health outcomes [8]. Sick leave may sometimes be warranted, but even short-term sick leave may increase the risk of future long-term absence [9]. Cardinal symptoms of depression and anxiety are avoidance and withdrawal, behaviours that may be reinforced by absence from social arenas such as the workplace. There is therefore broad agreement that treatment for patients on sick leave due to depression and anxiety should aim to help patients remain at work, or in the case of sick leave, return to work (RTW) [10].

Cognitive behavioural therapy (CBT) is effective for treating depression and anxiety symptoms [11], but its impact on work status is most likely limited. One meta-analysis from 2017 found that standard CBT without work-focused interventions had no impact on the work status of patients with depression and anxiety [12]. This is consistent with an evaluation of mental health and work conducted by the Organisation for Economic Co-operation and Development (OECD): in treatment for sick-listed workers, work status is neither a core competency, priority or outcome measure in traditional psychotherapy [13]. Results from a recent randomised controlled trial evaluating “Prompt Mental Health Care”, a programme for treating common mental disorders, are consistent with this conclusion. In this study, CBT was more effective than treatment as usual for reducing symptoms of anxiety and depression, but did not improve rates of return to work [14].

Attempts have thus been made to integrate work-focused interventions with standard treatment of common mental disorders [15]. To our knowledge, one of the first RCTs on treatment for patients with common mental disorders that include RTW as an outcome measure was conducted in the Netherlands in 2006, concluding that work-focused CBT (W-CBT) lead to earlier RTW [16]. Further research in the Netherlands found that adding work-focused interventions to treatment yielded effective symptom reduction, reduced the number of working days lost and had a high probability of being cost-effective [17]. A 2012 comparative outcome study found that patients who received W-CBT returned to work faster than participants receiving only CBT [18]. Since then, further support for W-CBT has been found in German and Swedish studies [19, 20]. Recently, reviews of the literature on work-focused treatment found strong evidence that CBT paired with work-focused interventions can reduce the duration of and costs due to sick leave, and recommends further implementation of such programmes [12].

Despite the encouraging results, combining psychotherapy with work-focused interventions is still in its infancy. A systematic review and meta-analysis from 2016 [21] indicated that there is a high degree of heterogeneity between studies. This is related to the type of therapies used, which work interventions are used, how they are implemented and how the outcomes are measured, making comparison difficult [21]. Additionally, a clear description of the therapy, the work focused interventions and adherence is often lacking [22]. This underlines the need for preregistered high-quality randomised controlled trials and publications of study protocols [22].

In addition to the need for more rigorous studies, there is also room for improving treatment outcomes [22]. The majority of the work-focused treatment studies have been modelled on CBT as the evidence for its efficacy in treating depression and anxiety symptoms is well explored [11]. The research in general shows significant improvement in symptoms in approximately 50% of patients but this masks considerable variability in outcomes across studies [23, 24]. Furthermore, a treatment where half of the patients do not get significantly better leaves considerable room for improvement. Follow-up of 1- and 2-year relapse rates show that 25–50% of patients treated for depression relapse, and the corresponding number for anxiety is about 15% [25, 26]. In recent years, metacognitive therapy (MCT) has shown excellent results in treating depression and anxiety [27]. Recent studies on MCT in Norway have shown recovery rates of 65% and 70% for anxiety and depression, with similar rates at 1 and 3-year follow-up [28–31]. In Denmark a comparison of MCT with CBT for depression showed that MCT was superior, achieving a 74% recovery rate compared with 52% in CBT [32]. A separate Norwegian study also showed that metacognitions, a central construct in MCT, may also predict work status [33]. This indicates that MCT is a very promising treatment which may represent a significant step forward in terms of recovery from depression and anxiety and may be more effective than existing approaches.

An observational study at Diakonhjemmet Hospital in Norway, which included work-focus interventions found that significantly more patients returned fully to work at end of treatment in the intervention group than in the wait-list group (41.4% versus 26.3%) [34]. The findings from this study are promising, warranting further exploration in a RCT.

To date, no study has investigated Metacognitive therapy (MCT) and work-focused interventions together. This waitlist RCT primarily evaluates the changes in symptoms of depression and anxiety, and percentage of sick leave, and secondarily whether the treatment is cost-effective. The main hypotheses to be tested are:

1. MCT plus work-focused interventions will be superior to the wait-list condition in reducing symptoms of anxiety and depression and the percentage of sick leave. Results will examine post-treatment and for the intervention group at 6 and 12 months follow-up
2. MCT plus work-focused interventions will be cost-effective when compared with the wait-list condition. Self-report data on the percentage of sick leave will examine post-treatment, 6 and 12 months follow-up, in addition with the national register data span from 24 months prior to treatment and 48 months after ending the treatment

## Methods

### Primary objectives

The primary objective of the study is to examine the impact of MCT plus work-focus interventions on symptoms of depression and anxiety and percentage of sick leave at end of treatment. Other measurement points will be included in secondary objectives. Self-report data will be collected at initial assessment, start and end of treatment as well as at 6 and 12 months follow-up. Data from the national registry related to percentage of sick leave is collected up to 24 months prior to and 48 months after treatment. A schematic overview of measurements and endpoints are included in Fig. 2. The following measures will be used:

1. Changes in the degree of sick leave recorded from National registries (time frame: from 2 years prior to intervention to 4 years after intervention).
2. Changes in the degree of sick leave from patient self-report (time frame: from pre-treatment, to post-treatment (12 weeks), and at 6 months and at 1 year follow-up).
3. Changes in anxiety symptoms as recorded by Beck Anxiety Inventory (BAI) [35] (time frame: from pre-treatment to post-treatment (12 weeks) and at 6 months and 1 year follow-up).
4. Changes in depressive symptoms measured by Beck Depression Inventory II (BDI-II) [36] (time frame: from pre-treatment to post-treatment (12 weeks) and at 6 months and 1 year follow-up).

BDI-II and BAI are self-reported measures. For the percentage of sick leave, both national registry data will be collected from the National Labour and Welfare administration in addition to self-report.

### Secondary objectives

The secondary objective of the study is to determine whether the treatment is cost-effective compared to the wait-list condition and to examine other exploratory outcome measures. The questionnaires will be collected at screening, first treatment session and last treatment session. Follow-up questionnaires will be collected at 6 and 12 months post-treatment. The exception is change in diagnoses which will be evaluated by clinical interview at screening, before the first treatment session and after the last treatment session.

Measurements for the study’s secondary objective:

1. Changes in quality of life as measured by the EQ-5D-5L [37]
2. Changes in metacognitions as measured by the Metacognitions Questionnaire 30 (MCQ-30) [38]
3. Changes in bullying and victimisation as measured by the Negative Acts Questionnaire [39].
4. Changes in work-related self-efficacy as measured by the Return to Work Self-Efficacy questionnaire (RTW-SE) [40].
5. Changes in resilience as measured by The Resilience Scale for Adults (RSA) [41]
6. Changes in subjective health complaints as measured by the Subjective Health Complaints questionnaire (SHC) [42].
7. Change in diagnoses as measured by the Mini-International Neuropsychiatric Interview (M.I.N.I.) [43].

### Other outcome measures

Secondary analysis related to the onset of COVID-19 (time frame: from pre-treatment to post-treatment (12 weeks) and 6 months and 1 year follow-up). Sub-analyses will take into consideration the onset of COVID-19 in Norway by the middle of March 2020. In addition to potentially influencing depression and anxiety symptoms, COVID-19 may have influenced the work situation for participants. Thus, it is important to explore if this is the case for those recruited to the project after the outbreak of the pandemic. The COVID-19 pandemic hit during the project, and it may have influenced initial levels of anxiety and/or depressive symptoms. We therefore think it would be important to explore if those included prior to and after the onset are systematically different regarding symptom severity. Also, the pandemic with a subsequent lock-down of many central society services affected many persons work situation. Many in service occupations lost their jobs and others were put on unpaid leave. Regarding the output variable return to work, it is important to check if many of the patients were in this situation. We therefore included a questionnaire that asks patients included during the Covid pandemic whether their working situation, sick leave or income was affected by the pandemic.

### Study design

The study is an RCT comparing MCT and work-focused interventions given immediately after screening (< 1 week after assessment) with a wait-list condition. We chose a waitlist design as a “no treatment” control group is not feasible due to ethical reasons, and comparison with another active treatment was not feasible due to practical reasons. The intervention will be given as manualised treatment based on diagnostic-specific MCT as specified in the manual from 2009 by Wells [44]. Work-focused interventions will be based on the work module used in Lagerveld’s study but adjusted for a Norwegian context and MCT [18].

**Fig. 1:**
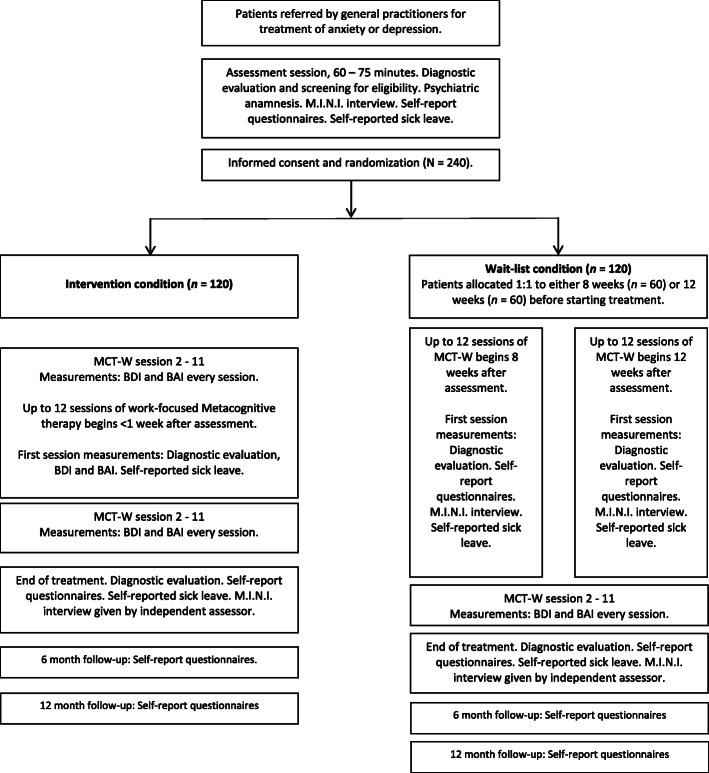
Summary flow chart. The self-report questionnaire packet contains *BAI* Beck’s Anxiety Inventory; *BDI-II* Beck’s Depression Inventory-II; *EQ-5D* Euro-Qol 5D; *RTW-SE* Return to Work Self-efficacy; *MCQ-30* Metacognitive Questionnaire 30; *SHC* Subjective Health Complaints; *RSA* Resilience Scale for Adults; *RTW-E* Return to Work Expectation; *SNAQ* Short Negative Acts Questionnaire; *NAQ-R* Negative Acts Questionnaire Revised; *AUDIT* Alcohol Use Disorders Identification Test; *SAPAS-SR* Standardised Assessment of Personality Self-report. In addition, registry data on sick leave will be collected from 2 years prior to 4 years post intervention

A summary flowchart of the procedure is found in Fig. 1. Treatment duration is up to weekly 12 sessions, but as the study takes place in a national health service outpatient clinic, the number of sessions may vary. The treatment primary outcome is measured at end of treatment or session 12 if treatment duration is longer, whichever comes first. In all, 240 patients who are on sick leave due to anxiety or depression at the time of enrolment will be recruited. The study uses a block randomisation with a stratification based on gender and degree of sick leave to ensure an equal balance of gender and sick leave across the conditions. Degree of sick leave indicates the percentage of ordinary working hours. Someone working half their normal number of hours would thus be on 50% sick leave. An equal number of patients are randomised to the treatment and wait-list condition. The waitlist condition group is subdivided into two equal groups that wait either 8 or 12 weeks before treatment, because in the sample there will be variation in the duration of the treatment intervention. The registry data will benchmark the current study against previous studies that have used CBT with regards to RTW. The maximum waiting time of 12 weeks is identical to the average waiting time at the clinic, to ensure that participation in the study does not delay treatment for patients. The reason for differentiating the waitlist condition is twofold. First, Norwegian law dictates that patients with moderate to severe depressive episodes should not wait longer than 8 weeks. Secondly, it is important to investigate if the duration of waiting in itself accounts for differences in change, e.g. whether shorter or longer waiting times improves outcomes.

### Eligibility criteria

Participants must:

1. Be adults of working age (18–67 years)
2. On sick leave either partial or full, due to
3. Anxiety and/or mild to moderate depressive disorders, assessed using MINI International Neuropsychiatric Interview,
4. Eligible for work-related sick leave payouts, and
5. Provide written consent

In addition to the inclusion criteria, participants will be excluded if they are:

1. Suffering from psychosis (i.e. bipolar disorder, schizophrenia or other psychotic disorders)
2. Engaged in active substance abuse
3. Suffer from cluster A or B personality disorder

### Recruitment process and context

Participants are recruited from the ordinary patient population of *Poliklinikken Raskere tilbake*, a psychiatric out-patient clinic at Diakonhjemmet Hospital in Oslo, Norway. The clinic was originally funded by the national programme “Faster Return” aimed at reducing sick leave and is a part of the national health service. The present study is part of NOR-WORK, the Norwegian studies of psychological treatments and work.

The Norwegian welfare system grants sick-listed employees compensation equivalent to their full salary from the first day of sick leave. This compensation is covered by the employer for the first 16 days of sick leave. The state then covers the compensation from day 17 and up to one full year of sick leave. If an employee is still not able to return to work after a full year on sick leave, they receive a different compensation package equivalent to 66% of their original pay.

Patients are referred by their general practitioners and receive self-report questionnaires by mail before attending a screening session with a clinical psychologist. The treatment is conducted in Norwegian, and the questionnaires are also written in Norwegian. Patients thus have to speak, read and write Norwegian in order to participate. The screening session lasts 60–75 min, and patients are diagnosed based on a clinical assessment which includes the MINI-International Neuropsychiatric Interview.

### Informed consent

Participants are presented with written information on the study and their potential participation by mail prior to their assessment session. The information states clearly the content and purpose of the study, and that any offer of treatment at the clinic is not contingent on participation in any ongoing trials. Patients who do not wish to participate in research, but are eligible for treatment at the clinic, will receive the same treatment from the same group of therapists as those who choose to enrol in the trial. The consent also states clearly what data will be collected, what it will be used for and for how long it will be stored. At the initial assessment session, the therapist also goes through the written consent with the patient, explains the content and answers any questions that the patient may have. Written consent is then signed by both patient and therapist before enrolment in the trial. The informed consent has been evaluated by a representative from the patient interest organisation “Mental Health” and approved by the relevant governing body, the Regional Committee for Medical and Health Research Ethics.

### Randomisation and blinding

After the initial screening session, eligible patients will be randomised using WebCRF, a web-based program provided by the Unit for Applied Clinical Research at the Norwegian University of Science and Technology (NTNU). The block randomisation is stratified on gender and degree of sick leave. The study will include 240 patients in all. Half of the patients will be allocated to the treatment condition (*n* = 120) and the other half to the wait-list conditions (*n* = 120). Half of the patients in the wait-list condition wait 8 weeks, the other half wait 12 weeks before commencing treatment. Because the treatment is offered in an ordinary outpatient clinic, the duration of the treatment will vary. To control for potential differences in treatment duration the wait-list condition also contained two different conditions.

Guidelines from the Norwegian Directorate for Health and Social Affairs state that patients who fulfil the criteria for a moderate depressive episode according to ICD-10 have the right to specialised health care within 8 weeks of referral [45, 46]. Thus, patients with this diagnosis will be allocated evenly between the treatment condition and the 8-week waiting condition. No patients with moderate depressive episode will be allocated to the 12-week waiting condition due to Norwegian law.

The randomisation procedure is performed by administrative personnel who otherwise are not involved in the patient’s treatment. Clinicians are not involved in the randomisation procedure. The initial assessment session is carried out by a different therapist than the one the patient sees for treatment. Blinding the treatment therapist to group allocation is not doable for logistical reasons (e.g. date of journal documents). Assessment of diagnosis at end of treatment using the Mini-International Diagnostic Interview is carried out by an independent clinician who has no prior contact with the patient.

### Outcome measures

#### Primary outcome measures

The primary outcome in the current study is as follows:

##### Beck Depression Inventory-II

BDI-II is a 21 item self-report measure of depression severity over the last 2 weeks. Each of the 21 items is scored from 0 to 3, giving a score range of 0–63. A higher score indicates more severe symptoms. The BDI-II has a Cronbach’s alpha of 0.94 and test-retest reliability of 0.93 [36]. In a previous Norwegian study with patients on sick due to common mental disorders, the Chronbach’s alpha for this measure was 0.89 [34].

##### Beck Anxiety Inventory

BAI is a self-report measure of anxiety severity over the last week. Like the BDI-II, it has 21 items that are scored from 0 to 3, giving a total score ranging from 0 to 63. Higher scores indicate more severe symptoms. The BAI has an alpha of 0.92 and a test-retest reliability of 0.75 [47]. The BAI had a Chronbach’s alpha of 0.90 in a previous Norwegian study of patients on sick leave due to common mental disorders [34].

##### Sick leave

**Fig. 2:**
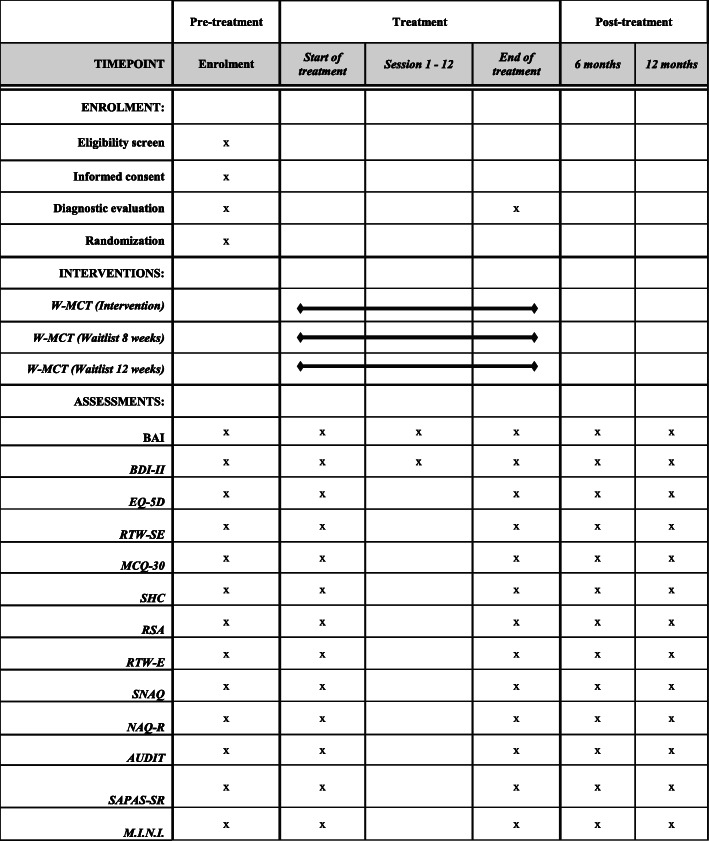
SPIRIT figure. Schedule of enrolment, interventions, and assessment*. Abbreviations: BAI* Beck’s Anxiety Inventory; *BDI-II* Beck’s Depression Inventory-II; *EQ-5D* Euro-Qol 5D; *RTW-SE* Return to Work Self-efficacy; *MCQ-30* Metacognitive Questionnaire 30; *SHC* Subjective Health Complaints; *RSA* Resilience Scale for Adults; *RTW-E* Return to Work Expectation; *SNAQ* Short Negative Acts Questionnaire; *NAQ-R* Negative Acts Questionnaire Revised; *AUDIT* Alcohol Use Disorders Identification Test; *SAPAS-SR* Standardised Assessment of Personality Self-report; *M.I.N.I* Mini-International Neuropsychiatric Interview

Self-reported percentage of sick leave will be collected pre- and post-treatment as well as 6 and 12 months follow-up. In addition, the study has permission to collect data from national register data on the percentage of sick leave up to 24 months prior to treatment and 48 months after treatment. Norway has extensive national registries for sick leave and benefit pay-outs, creating a unique opportunity to evaluate treatment effects in this regard. The 2 years prior to treatment will serve to establish a baseline. Figure 2 shows the schedule for the collection of self-report measures.

#### Secondary outcome measures

##### EuroQol five-dimensional descriptive system

The EQ-5D 5L is generic a self-report measure for describing and valuing health status. Health is described in five dimensions (5D): Mobility, Self-Care, Usual Activities, Pain/Discomfort and Anxiety/Depression. Each dimension has five levels (5L) scoring from no to extreme problems. The EQ-5D-5L also includes a rating of own health on a 0–100 visual analogue scale. Quality-adjusted life years (QALY) will be estimated from the EQ-5D-5L using the English utility tariff, which will support the cost-effectiveness analysis [37]. The use of the EQ-5D-5L for evaluating health technology in Norway is recommended by the Norwegian Medicines Agency [48].

##### Return to Work Self-Efficacy Scale

RTW-SE is an 11 item self-report questionnaire of self-efficacy in returning to work. Respondents are asked how they would deal with overcoming obstacles and respond to statements such as “I will be able to cope with potential problems at work” or “I will be able to manage set-back” using a six-point Likert scale. Both baseline score and change in the score during treatment have been shown to be robust predictors of return to work [49]. The scale has also been validated in a Norwegian sample [50].

##### Metacognitions Questionnaire 30

MCQ-30 measures metacognitive beliefs in a 30-item self-report questionnaire. Beliefs are scored across five factors: cognitive confidence, positive beliefs about worry, cognitive self-consciousness, negative beliefs about the uncontrollability of thoughts and danger and beliefs about the need to control thoughts. Higher scores indicate higher levels of maladaptive metacognition. The validity and reliability of MCQ-30 is well-established in adults [38], including in Norwegian samples with depression and anxiety [51].

##### Subjective Health Complaints

SHC is a self-report questionnaire measuring subjective health complaints along five factors: musculoskeletal pain, pseudo-neurology, gastrointestinal problems, allergy and flu. The aim of the SHC is to provide a simple measure of the most common complaints seen by general practitioners, and it was developed and validated using Norwegian patient samples [42].

##### The Resilience Scale for Adults

RSA is a self-report questionnaire measuring self-efficacy across five subscales: personal competence, social competence, family coherence, social support and personal structure. Respondents answer 33 items using a seven-point Likert scale. The RSA scale is a valid and reliable measurement of protective factors important to regain and maintain mental health. Three of the RSA subscales have previously been shown to be a significant predictor of employment status in a Norwegian sample [41].

##### Return to Work Expectation

RTW-expectation is a single-item scale measured by participant response to the following statement “I expect to return to work within the next few weeks” on a five-point Likert scale. RTW-expectation has been used in several previous studies and has been shown to predict future benefit recipiency in a Norwegian sample of patients with depression and anxiety struggling with work participation [52].

##### The Negative Acts Questionnaire-revised

NAQ-R is a 22 item self-report questionnaire developed to measure exposure to bullying in the workplace. It has a Cronbach’s alpha for the 22 items of .90, indicating excellent internal consistency, and has been shown to be valid in a Norwegian sample [39].

##### Alcohol Use Disorder Identification Test

AUDIT is a questionnaire used to screen for alcohol abuse. It has ten questions and yields a score range of 0–40 with higher scores indicating more severe problems [53]. It has been shown to have good psychometric properties including high test-retest reliability across multiple studies [54].

##### The Standardised Assessment of Personality Abbreviated Scale – Self report

SAPAS-SR is an 8 item self-report questionnaire. It has shown good sensitivity (80%) and specificity (83%) in identifying personality disorders in clinical populations [55].

### Intervention—metacognitive therapy and work-focused module

The trial uses MCT according to the manual [31] and work-focused interventions according to the work-module that has shown promising outcomes in a previous observational study [34]. The MCT interventions and the work-focused interventions are provided in the same session by the same therapist.

#### Work-focused module

The guiding principles in the work module form a checklist to ensure that work-focus is maintained throughout the intervention. Crucially, sick leave and potential RTW is addressed from the very onset of treatment. Guiding principles are as follows:

1. Map the working situation, analyse job type, working conditions, relations and how symptoms affect the patient at work.
2. Provide psychoeducation on work and mental health, including pros and cons of sick leave, e.g. risk of increased isolation and withdrawal.
3. Map degree of sick leave and generate a gradual RTW plan. Explore the possibility of gradual RTW from the onset of therapy and communicate this to the patient’s general practitioner (GP). In the Norwegian system, GPs are responsible for sick leave, and communication with GPs is therefore an integral part of the module. Therapists are encouraged to update GPs on treatment progress, and in cooperation with patients, discuss the duration and degree of sick leave.
4. Explore and map potential barriers for return to work, e.g. bullying, serious work conflicts, serious somatic disorder.
5. Encourage patient to establish a dialogue with the workplace, by generating an information strategy with the patient on how to discuss their absence and symptoms in the workplace, and with whom.
6. Explore the need for workplace adjustments. Ask the patient about possibilities to adjust the working situation for a period.

#### Metacognitive therapy

MCT is based on the Self-Regulatory Executive Function (S-REF) model which postulates how human information processing contributes to the development and maintenance of mental disorders [56, 57]. It describes how maladaptation in attentional focus and the regulation of thought processes of rumination, worry and use of maladaptive coping are derived from biassed metacognitions [44]. This maladaptive response is labelled the cognitive attentional syndrome (CAS). The CAS consists of persistent worry and rumination, threat monitoring and ineffective maladaptive coping strategies [58]. The CAS is maintained by erroneous meta-beliefs about thinking. These maladaptive metacognitions are internal information processes that control, monitor and appraise thinking. MCT indicates that there are both positive and negative metacognitive beliefs that influence information processing and choice of coping strategies. MCT states that changing these metacognitions will alleviate mental health problems. Counteracting maladaptive coping like avoidance and isolation is made explicit when drafting the initial case formulation in cooperation with the patient. The therapist uses the case formulation as a “roadmap” and reference point for the duration of the treatment, targeting metacognitions. MCT targets worry and rumination, which are cognitive processes that are also central for RTW [58, 59]. For instance, patients are less likely to RTW if they worry more about their condition, if they believe that their health issues are less treatable, less controllable, and more serious irrespective of actual severity [60, 61]. The same is true for negative expectations about work, such as believing that symptoms will worsen if one returns to work [52], in MCT terms this is considered and labelled worry. Patients often evaluate their situation more negatively than e.g. occupational physicians do [62], this may be attributable to the patients perseverative negative thinking labelled rumination in MCT. Targeting such maladaptive thinking processes and coping strategies is a central part of MCT.

The sequence in MCT treatment is as follows [38]:

1. Case conceptualisation, mapping symptoms and triggers.
2. Socialise the patient to the MCT treatment model.
3. Uncover and learn to recognise triggers for rumination or worry.
4. Attention training, learning to shift attention in ways that are specifically designed to modify metacognitions.
5. Challenge beliefs about uncontrollability of rumination and worry.
6. Challenge other negative metacognitive beliefs.
7. Challenge positive metacognitive beliefs.
8. Eliminate maladaptive coping strategies and reinforcing new plans for regulating actions.
9. Relapse prevention. Explore remaining worry, rumination or maladaptive coping strategies or impairments the patient may have.

The work-focused interventions in the present study are in line with the rationale and treatment protocol of MCT. It is not intended to be a stand-alone treatment programme and is linked into the case formulation of MCT whenever possible.

Anamneses contain information on work, maladaptive thought processes and maladaptive coping strategies are integrated in the MCT case formulation in line with the MCT treatment protocol. Furthermore, the emphasis is on exploring the CAS, such as worry, rumination and maladaptive coping strategies, and how these MCT constructs manifest in relation to work. Examples of maladaptive coping strategies at work may be avoidance, suppression and distraction as well as worrying prior to work tasks, rumination in hindsight or potential threat monitoring during work-related tasks.

### Treatment adherence and fidelity

Treatment is provided by a group of ten certified metacognitive therapists, educated at the MCT Institute in Manchester under the supervision of Professor Adrian Wells and Professor Hans M. Nordahl. The small therapist group size facilitates treatment integrity, and all therapists are trained in work-focused interventions. All therapists participate in supervision overseen by Professor Odin Hjemdal, who is also the project leader. Therapists participate in weekly group supervision, monthly individual supervision and use video recordings of sessions to monitor progress. A random subset of therapy sessions will be recorded, subject to patient consent. Therapists report intervention adherence through filling out checklists at end of treatment for each patient. Adherence is also secured through written treatment logs which are part of the standard MCT protocol [38].

### Control condition and concomitant care

The control condition in the trial is a wait-list where patients receive no intervention. During the waitlist condition and the intervention, medication is administered by the participant GP in accordance with national guidelines. Use of medication and health services are recorded at assessment, start of treatment and end of treatment through self-report forms. No other psychotherapy will be allowed during the 12-week treatment period of the trial. However, the frequency of accessing additional therapy during the follow-up period will be assessed. Somatic health status, including any somatic diagnoses, is recorded by self-report and will be reported as part of patient characteristics in all publications.

### Safety reporting

No adverse consequences are expected. However, in the event of any adverse effect, patients will be treated in accordance with national health care guidelines to ensure optimal care. Any patient can discontinue participation in the trial at any time without disclosing their reasons. Any adverse events will be recorded and reported in accordance with Good Clinical Practice and national patient safety regulations. The clinic in which the study is taking place is part of the national health service, and the study is subject to all rules and regulations that regulate specialised health care, including mandatory reporting of adverse events.

The study will be monitored by a trial research committee and an international advisory board. Both are involved in overseeing the trial and assuring that research ethics and principals for good research are followed.

### Retention and follow-up

We will strive to collect data on all patients, regardless of whether they complete treatment or not. The clinic has collected self-report questionnaires from patients for clinical use since its inception in 2007, and the infrastructure for data collection is well-established. For the post-treatment follow-up at 6, and 12 months, self-report measures will be mailed to the patient. Patients will also receive a reminder per SMS and a follow-up phone call. Data on sick leave will be collected from national registries and is thus less vulnerable to loss to follow-up.

### Sample size estimate

Sick leave will be operationalised as a percentage of sick leave collected both from the national registry and self-report. To evaluate the intervention versus wait-list condition, the percentage of sick leave status of the intervention group will be compared at end of treatment to the status of the control group at the end of the waiting period. The minimum expected effect is that of the treatment reported in the naturalistic follow-up study at the same clinic [56]. Data from this study showed a self-reported full RTW response rate of 0.41 in the intervention group versus 0.26 in the wait-list control group, with a pooled standard deviation of 0.4 [34]. Given this result, to detect differences in a dichotomous outcome variable we would need approximately 120 in each treatment group to have an 80% chance of detecting a significant difference at a two-sided significance level of 0.05 [63]. For the anxiety and depressive symptoms, detecting a 5 point difference between intervention and waitlist control group using a pooled standard deviation of 6.95, we need approximately 31 participants in each of the group in order to have an 80% chance of detecting a significant difference at a two-sided significance level of 0.05 [28, 64].

### Data analysis and data management

Data analysis will be conducted in accordance with a pre-specified data-analysis plan. All data will be analysed based on an intention-to-treat (ITT) approach, and analyses will include all randomised patients. The primary outcomes are continuous measures in a repeated design, therefore linear mixed-model analysis (LMM) will be run. For effects on depression and anxiety, reliable clinical change index and Cohen’s *d* for effect size will be reported, the difference between groups will be analysed using mixed model ANOVA.

All data is recorded and managed in accordance with national health service guidelines. Access to data is managed by Diakonhjemmet Hospital.

### Health economic analyses

The primary cost-effectiveness analysis will estimate total costs and QALYs for the 12-month follow-up period of the trial, using an intention-to-treat approach. Service use is not being collected in the trial and the cost-effective analysis will therefore be integrated within the trial and will include intervention costs alone. These will be estimated per patient and will include the cost of staff time, overhead costs and any other materials needed to deliver MCT. QALYs from baseline to follow-up will be estimated using the EQ-5D-5L. A Norwegian value set does not currently exist, and the English value set will be used [65]. EQ-5D-5L responses and index values will be reported and compared to the Norwegian general population norms [66].

Regression analysis will be used to estimate net costs and net QALYs and these estimates will be bootstrapped to generate 10,000 net pairs of costs and QALYs to inform the probability of cost-effectiveness. The incremental cost-effectiveness ratio (ICER), a joint measure of cost and health benefit, will be the key outcome, and a cost-effectiveness plane and cost-effectiveness acceptability curve will be plotted.

As the trial is not collecting service use to inform wider healthcare costs and the time horizon is limited, the economic evaluation will be furthered with the development of a decision analytic economic model which will synthesise trial data with the published literature. The model structure will be drafted considering the existing literature and the trial design, following which it will be reviewed and validated with clinicians. A literature review will be used to identify data not available within the trial (e.g. broader healthcare resource use and costs). As with the trial-based analysis, the key outcome will be the ICER (per QALY).

Guidance of the methods for economic models will be followed and the economic evaluation will adhere to reporting guidelines. Sensitivity analysis will explore uncertainty, in particular, the impact of using alternative measures of benefit, perspectives of cost and time horizons.

### Dissemination

A trial publication board will meet regularly, and the board sets the publication policy for the trial. Currently, three Ph.D. candidates are expected to utilise data from the study and publish articles in peer-reviewed journals.

## Discussion

The aim of this trial is to investigate the impact of MCT and focused interventions on sick leave and symptoms of depression and anxiety, and whether this treatment is cost-effective. This is the first RCT to examine the effect of MCT and adapted work-focused intervention, contributing high-quality evidence on a novel approach aimed at a significant public health issue. As the study will include 240 patients, this is a fairly large clinical trial, and the long follow-up period will yield a considerable amount of outcome data.

A recurrent issue in the literature is that therapy and work interventions vary greatly between studies and that the therapy and the interventions are not adequately described, making comparison and replication difficult. The present protocol attempts to address this issue by describing the content and structure of a well-defined therapy and work intervention in detail.

There are many factors contributing to sick leave, and any given treatment is unlikely to address all issues for all patients. The large amount of outcome data in the present study can help to describe some associations between contributing factors, and thus inform future research questions. Similarly, registry data collected up to 4 years after completion of the trial will give reliable data on this patient group beyond the immediate end of treatment.

The relatively short wait-list period in the trial design may pose a challenge to detecting differences in outcomes. The Norwegian regulation regarding moderate depression stipulates that 8 weeks is the maximum delay before treatment onset after diagnostic evaluations. The intention in the regulation is to assure that patients with moderate depression experience as little delay in the onset of treatment as possible. This regulation may be specific to Norway and may complicate comparisons with results from similar studies from other countries. It is a limitation with the study, however, the setting of research project is a regular outpatient clinic, and regulations of clinical activity also has implication for research. A potential strength of the project precisely related to the setting is the relatively high ecological validity. On this note, it is worth mentioning that our previous observational study had a similar time-frame and showed good results for sick leave and symptom reduction in the intervention versus wait-list condition [34]. It is also the case that the current trial is monocentric. For the future, it would be of interest to replicate the study in a multicentre trial. Lastly, the economic impact of mental health problems and reduced employment is a significant societal expense. The result of the trial’s economic analyses can help inform policy decisions and clinical practice. As the ability to work is an important determinant of health and well-being, we believe patients suffering from depression and anxiety will benefit from expanding the evidence base on specialised care that includes interventions aimed at work-functioning.

## Trial status

The trial is carried out by Diakonhjemmet Hospital in cooperation with the Norwegian University of Science and Technology with additional funding from Sitftelsen Dam and the Norwegian Southern and Eastern Regional Health Authority. Recruitment began 1 January 2018 and was completed in May 2021. This protocol has been written to comply with the Standard Protocol Items: Recommendations for Interventional Trials (SPIRIT) statement (see Additional file 1 “SPIRIT checklist”). The final report will follow the Consolidated Standards for Reporting Trials (CONSORT) statement. Protocol version: Original, issued 25 August 2020.

## Supplementary Information

**Additional file 1.** SPIRIT checklist.

## Data Availability

Data management including access is handled by Diakonhjemmet Hospital.

## Abbreviations

AUDIT: Alcohol Use Disorders Identification Test
BAI: Beck’s Anxiety Inventory
BDI-II: Beck’s Depression Inventory-II
CAS: Cognitive attentional syndrome
CBT: Cognitive behavioural therapy
EQ-5D: Euro-Qol 5D
ICER: Incremental cost-effectiveness ratio
M.I.N.I: Mini-International Neuropsychiatric Interview
MCQ-30: Metacognitive Questionnaire 30
MCT: Metacognitive therapy
NAQ-R: Negative Acts Questionnaire Revised
NTNU: Norwegian University of Science and Technology
QALY: Quality-adjusted life year
RCT: Randomised controlled trial
RSA: Resilience Scale for Adults
RTW: Return to work
RTW-E: Return to Work Expectation
RTW-SE: Return to Work Self-efficacy
SAPAS-SR: Standardised Assessment of Personality Self-report
SHC: Subjective Health Complaints
SNAQ: Short Negative Acts Questionnaire
W-CBT: Work-focused cognitive behavioural therapy
MCT: Metacognitive therapy
