# Correction to: Metacognitive therapy and work-focused interventions for patients on sick leave due to anxiety and depression: study protocol for a randomised controlled wait-list trial

**DOI:** 10.1186/s13063-021-05933-y

**Published:** 2021-12-19

**Authors:** Kenneth Sandin, Ragne G. H. Gjengedal, Kåre Osnes, Marit Hannisdal, Torkil Berge, Jonas S. R. Leversen, Lars G. Røv, Silje Endresen Reme, Suzanne Lagerveld, Roland Blonk, Hans M. Nordahl, Gemma Shields, Adrian Wells, Odin Hjemdal

**Affiliations:** 1 Division of Mental Health and Substance Abuse, Diakonhjemmet Hospital, Postboks 23 Vinderen, 0319 Oslo, Norway; 2 Department of Psychology, Norwegian University of Science and Technology, NO-7491 Trondheim, Norway; 3 Department of Psychology, Faculty of Social Sciences, University of Oslo, Oslo, Norway; 4 Dutch Institute for Employee Benefit Schemes (UWV), Amsterdam, The Netherlands; 5 TNO Leiden, Leiden, The Netherlands; 6 Faculty of Medicine and Health Sciences, Norwegian University of Science and Technology, Trondheim, Norway; 7 Division of Population Health, Health Services Research, and Primary Care, University of Manchester, Manchester, UK; 8 Faculty of Biology Medicine and Health, The University of Manchester, Manchester, UK

**Correction to: Trials 22, 854 (2021)**

**https://doi.org/10.1186/s13063-021-05822-4**

Following the publication of the original article [1], we were notified of the below corrections:

- In the Abstract, under “Methods”, the final sentence originally began with “Symptoms of scores...” and was corrected to “Symptoms of depression and anxiety...”
- Under Background on page 3, the second sentence of the final paragraph: “This waitlist RCT primarily evaluates the changes in symptoms of depression and anxiety depression and anxiety percentage of sick leave...” was corrected to: “This waitlist RCT primarily evaluates the changes in symptoms of depression and anxiety, depression and anxiety percentage of sick leave...”

The original article has been corrected.
